# The association of *APOE *genotype and cognitive decline in interaction with risk factors in a 65–69 year old community sample

**DOI:** 10.1186/1471-2318-8-14

**Published:** 2008-07-14

**Authors:** Helen Christensen, Philip J Batterham, Andrew J Mackinnon, Anthony F Jorm, Holly A Mack, Karen A Mather, Kaarin J Anstey, Perminder S Sachdev, Simon Easteal

**Affiliations:** 1 The Australian National University, Canberra, Australia; 2 The University of Melbourne, Melbourne, Australia; 3 School of Psychiatry, University of New South Wales, Sydney, Australia; 4 Neuropsychiatric Institute, the Prince of Wales Hospital, Sydney, Australia

## Abstract

**Background:**

While the evidence of an association between the apolipoprotein E (*APOE*) *E4 allele and Alzheimer's disease is very strong, the effect of the *E4 allele on cognitive decline in the general population is more equivocal. A cross-sectional study on the lifespan effects of the *E4 allele [1] failed to find any effect of the *E4 allele on cognitive performance at ages 20–24, 40–44 or 60–64 years.

**Methods:**

In this four year follow-up study, we reexamine the effect of *E4 in the sample of 2,021 individuals, now aged 65–69 years.

**Results:**

Performance on the Mini-Mental State Examination (MMSE) was significantly poorer for *E4 homozygotes than heterozygotes or non-carriers. The effects of the *E4 genotype on cognitive decline over four years were found on the MMSE and Symbol-Digit Modalities test but only when controlling for risk factors such as head injury and education. Analyses were repeated with the exclusion of participants diagnosed with a mild cognitive disorder, with little change.

**Conclusion:**

It is possible that *E4 carriers become vulnerable to greater cognitive decline in the presence of other risk factors at 65–69 years of age.

## Background

There is clear evidence that the presence of the apolipoprotein *E4 *allele (APOE *E4) is associated with increased risk of Alzheimer's Disease (AD). Forty to forty-five percent of individuals diagnosed with Alzheimer's type dementia have at least one *E4 allele, compared with approximately 15% of the American population [2,3]. Recent evidence also suggests that the risk of dementia is increased synergistically in APOE *E4 carriers exposed to additional health risks, such as head trauma, high alcohol use [1,4,5], comorbid ischemic cerebrovascular disease [6], or a previous stroke, [6,7].

An important question to address is whether APOE *E4 carrier status is associated with more rapid cognitive decline in the general population when those with either dementia or preclinical dementia are excluded. To date, evidence generally supports the finding that cognitive decline is more rapid for *E4 carriers compared to non-carriers in general population samples [8-10]. However, it is not clear at what age the effects of APOE genotype emerge. There have been relatively few prospective longitudinal studies of midlife and young-old adult age groups. One exception is a study by Greenwood and colleagues [11] which reported that the *E4 allele was associated with greater decline on a test of visual attention in a sample aged 50 years and older. Seventy four of the 94 participants in the study had first degree relatives with AD, resulting in a sample likely to be sensitive to early effects of *APOE*, and also likely to include a number of individuals prodromal for dementia. A second study [12] replicated the findings in a sample of 177 individuals (mean age of 59 years), 80% of whom had a first degree relative with AD. In the second study, the authors found effects on memory for location and working memory, with the effects being subtle and manifesting under conditions of high processing demand. They concluded that the *APOE *genotype "exerts effects on specific components of cognition in midlife" (p. 207). This conclusion concurs with recent research reporting that the deposition of amyloid beta in the brain appears to increase around this age period. Amyloid beta protein (Aβ1–42) in the cerebrospinal fluid decreases at about 60 years in both *E4 carriers and non-carriers, a finding which implies that more amyloid is being deposited in the brain at this time, with the rate being greater in *E4 carriers [13].

However, one of the largest studies of *APOE *including a cohort of 60–64 year old individuals [1] failed to find an effect of *APOE *on cognitive functioning. Six thousand five hundred and sixty people aged 20–24, 40–44 and 60–64 years were studied using cross-sectional data. Age differences in cognitive functioning were observed among the cohort groups, but no difference in cognitive performance as a function of APOE *E4 status was observed in any of the three age cohorts. We emphasized that these analyses needed to be repeated as our samples aged.

The present study reports Wave 2 data from the 60–64 year old cohort (now aged 65–69 years) examined four years later. Given that the sample may be at a critical age for the onset of decline, we hypothesized that the rate of cognitive decline over the four years from Wave 1 to Wave 2 would be greater for APOE *E4 carriers. Measures of memory, speed and working memory were taken since these are sensitive to cognitive aging and are early markers of the development of dementia [14-16]. Given its epidemiological significance and its widespread use as a screening tool for early cognitive decline, we also extended our previous analysis to examine the effect of APOE *E4 on the Mini-Mental State Examination, MMSE [17], a brief screening test for dementia. Moreover, we predicted that the effect of APOE *E4 was most likely to be observed in interaction with other risk factors associated with AD. We investigated head injury, a history of stroke or vascular disease, high past or current alcohol consumption, low educational status and low pre-morbid intelligence as risk factors (see above). Additional support for the importance of these risk factors comes from a review [18] confirming the importance of *APOE *in the brain's response to injury, and that carriers of the *E4 allele are more vulnerable to the effects of head injury. With respect to education and premorbid intelligence, the risk of more rapid memory decline is greater in *E4 carriers with less than 10 years education than those with more years [19]. The analyses were repeated excluding individuals with any indication of mild cognitive decline based on a clinical assessment.

## Methods

### Participants

The sample comes from the PATH Through Life Project [20], a large community survey concerned with the health and well-being of people who are 20–24, 40–44, and 60–64 years of age. Participants were sampled from the electoral rolls for the city of Canberra, Australia, and in the neighboring town of Queanbeyan. Registration on the electoral roll is compulsory for Australian citizens. Each cohort is being followed up every 4 years over a total period of 20 years. Results presented here concern the first two waves of interviews with the 60 + cohort (conducted in 2001–2002 and 2005–2006). Letters were sent to 4,832 persons 60–64 years of age inviting them to participate in the PATH study. Non-participants included 34 people out of the required age range, 182 had moved, 28 were dead, 209 could not be found, 1,827 refused or their English was too poor to allow an interview, and 2,551 were interviewed (58.3% of those found and in age range). The gender breakdown of the sample was 1,319 men and 1,232 women.

At the second wave, 2,222 of the 60s cohort (87% follow up rate) were interviewed. Of the 770 participants who were not interviewed at the second wave, 234 refused or were unable to be interviewed due to medical reasons, 25 could not be located and 70 died between the two waves. Individuals of non-Caucasian background (n = 89) and those who were not genotyped (n = 112) were excluded in these analyses, leaving a total of 2,021 participants in the final sample.

### Survey procedure

Persons selected at random from the electoral roll were sent a letter informing them of the survey and that an interviewer would contact them soon to see if they wanted to participate. If a person agreed to participate, the interviewer arranged to meet them at some convenient location, usually the participant's home or the Centre for Mental Health Research at the Australian National University. Most of the interview was self-completed on a palmtop or laptop computer. However, testing by the interviewer was required for the physical tests, for some of the cognitive tests, and for a cheek swab from which DNA could be extracted.

### Ethics approval

Ethics approval was obtained from the Australian National University's Human Research Ethics Committee.

### Genotyping

Genomic DNA was extracted from cheek swabs using Qiagen DNA Blood kits (#51162; Qiagen Inc., Valencia, CA, USA). To identify the six *APOE *genotypes comprising the APOE *E2, *E3 and *E4 alleles, two single nucleotide polymorphisms (SNPs) were assayed using the TaqMan method [Applied Biosystems Inc. (ABI), Foster City, CA, USA]. SNP-specific primers and probes were designed by ABI (TaqMan genotyping assays) and assays were performed according to the manufacturer's instructions in 5 μl total volumes in 384-well plates. The polymorphisms distinguish the *E2 allele from the *E3 and *E4 alleles at amino acid position 158 (NCBI *rs7412*) and the *E4 allele from the *E2 and *E3 alleles at amino acid position 112 (NCBI *rs429358*).

TaqMan real-time polymerase chain reaction assays (PCR) were performed in an ABI 7900 HT machine, using a cycling program of: 95°C for 10 min; 40 cycles of 95°C for 15 sec and 60°C for 1 min. Six positive controls, one for each genotype, and one negative control (water) were included in each plate and were consistently called correctly. The genotypes of the six positive controls were confirmed by *Cfo1 *restriction fragment length analysis, following PCR amplification of part of the *APOE *gene [21].

Allelic frequencies were consistent with Hardy-Weinberg expectations for the PATH 60+ age cohort for both Wave 1 (*rs7412*: χ^2 ^= 1.631, *df *= 1, *p *> .20, *n *= 2,281; *rs429358*: χ^2 ^= 0.002, *df *= 1, *p *> .97, *n *= 2,281) and Wave 2 (*rs7412*: χ^2 ^= 2.140, *df *= 1, *p *> .14, *n *= 2,021; *rs429358*: χ^2 ^= .646, *df *= 1, *p *> .42, *n *= 2,021). For the purposes of this analysis, participants were classified into three groups: *E4-/*E4- (no *E4 alelle), *E4+/*E4- (heterozygous for *E4) and *E4+/*E4+ (homozygous for *E4).

### Predictor or control variables

#### Education

Educational attainment was measured using six questions concerning the full spectrum of past and current primary (elementary), secondary and tertiary educational attainment. Responses to these questions were coded into a single measure corresponding to the number of years of education. For the purposes of the analyses, education was then categorized into four groups: 0–12 years, 13 years (i.e., high school), 14–15 years, and 16 years or more.

#### Head injury

Participants were classified as having head trauma if they responded positively to a question asking whether they had ever had a serious head injury that had caused them to become unconscious, at either Wave 1 or Wave 2 [22].

#### Hazardous or harmful alcohol consumption

Past alcohol consumption was measured using two items regarding the frequency and amount of alcohol consumption at the time the participant was drinking at their highest level. Respondents with potentially hazardous or harmful levels of drinking were identified as those who averaged more than 14 standard drinks per week. Current alcohol consumption was measured with the Alcohol Use Disorders Identification Test (AUDIT) [23]. Respondents who had a total AUDIT score of eight or higher were considered to be currently consuming harmful or hazardous levels of alcohol [23].

#### Premorbid intelligence

Intelligence was estimated using lexical decision performance on the Spot-the-Word Test Version A (STW), which asks participants to choose the real words from 60 pairs of words and nonsense words [24].

#### Stroke history

History of stroke was ascertained using one question, "Have you ever suffered a stroke, ministroke or TIA (Transient Ischemic Attack)?"

#### Current hypertension

Blood pressure was measured twice during the interview. Readings for diastolic and for systolic pressure were averaged for each interview. Hypertension was defined as having a mean systolic blood pressure ≥ 140, a mean diastolic blood pressure ≥ 90 at Wave 1 or as taking medication for hypertension.

### Cognitive tests

#### Speed

Mental speed was measured with the Symbol-Digit Modalities Test, which asks the participant to substitute as many digits for symbols as possible in 90 seconds [25].

#### Reaction time (RT)

Reaction time (RT) was tested using a small box held with both hands, with left and right buttons at the top to be depressed by the index fingers [26]. There were four blocks of 20 trials measuring simple reaction time (SRT), followed by two blocks of 20 trials measuring choice reaction time (CRT). Means were calculated after removing outliers. This was done by first eliminating any values under 100 ms or over 3,000 ms. Next, means and standard deviations were calculated for each individual for each block, and values outside three standard deviations from the individual's block mean were eliminated [26].

#### Memory

Immediate and delayed recall were assessed with the first trial of the California Verbal Learning Test [27], which involves recalling a list of 16 nouns. The interval between immediate and delayed recall was occupied by a test of grip strength.

#### Working memory

Working memory was assessed with the Digits Backwards subtest of the Wechsler Memory Scale [28], which presents participants with series of digits at the rate of one per second and asks them to repeat the digits backwards.

#### General cognitive impairment

The 11 item Mini-Mental State Examination (MMSE) [17] was administered primarily for screening purposes. However, the MMSE provides a reliable measure of mental status, covering aspects of cognition (in particular, orientation to time and place) that were not measured by the other cognitive tests.

Cognitive change scores were calculated for each test by subtracting Wave 1 scores from Wave 2 scores.

### Clinical assessment

At each wave, participants who scored below a predetermined cut-off on a screening battery were assessed for clinical diagnoses. Participants from the full cohort were selected for clinical assessment if they had any of the following: (1) a Mini-Mental State Examination (MMSE) score ≤ 25; (2) a score below the 5th percentile score on immediate or delayed recall of the California Verbal Learning Test (immediate or delayed score of <4 and <2, respectively), or (3) a score below the 5th percentile score for Wave 1 on two or more of the following tests: Symbol-Digit Modalities Test (<33), Purdue Pegboard with both hands (Wave 1: <8; Wave 2 <7) or reaction time (third set of 20 trials; Wave 1: > 310 ms; Wave 2: > 378 ms).

The clinical assessment in the PATH study has been described previously [29]. It involved a Structured Clinical Assessment for Dementia by one of two physicians which included a neuropsychological assessment and the Clinical Dementia Rating Scale. Where possible, an informant interview was undertaken. Diagnoses were made by consensus clinical judgment rather than by an algorithm according to criteria for diagnoses of Mild Cognitive Impairment [30], Age Associated Memory Impairment [31], Age Associated Cognitive Decline [32], or Mild Neurocognitive Disorder [33]. DSM-IV criteria were used to assess dementia [33].

### Analyses

A series of bivariate models were examined for the effect of *APOE *genotype, other risk factors, and *APOE *genotype by risk factor interactions on cognitive decline for each of the seven cognitive tasks. Multivariate ANOVA models for each cognitive change score were constructed, simultaneously including *APOE *genotype and any risk factors found to predict cognitive decline in association with genotype. Participants who did not complete a particular cognitive test at either Wave 1 or Wave 2 were excluded from the bivariate and multivariate analyses for that test. Sample sizes for bivariate and multivariate models were *n *= 1953 for SDMT, *n *= 1961 for immediate recall, *n *= 1961 for delayed recall, *n *= 1941 for digits backwards, *n *= 1953 for MMSE, *n *= 1877 for simple RT, and *n *= 1860 for choice RT. The reduced samples were due to a combination of missing premorbid intelligence data (24 cases) and missing cognitive test data (range: 36–137 cases). Analyses were repeated with the exclusion of those who were found to have any of the clinical diagnoses at either measurement point (*n *= 126). A significance level of .05 was used for individual analyses.

## Results

### APOE Genotyping

The genotyping results for this sample have been described previously in Jorm et al. [1]. In the 60+ sample of 2,021 participants, there were 1473 (73%) *E4 non-carriers, 510 heterozygous for *E4 (25%) and 38 (2%) homozygous for *E4.

### Demographics, risk factors and cognitive change as a function of APOE

Tables 1 and 2 show demographics, risk factors, cognitive performance (Wave 2) and change scores (Wave 2 scores minus Wave 1 scores) as a function of *APOE *genotype. The p-values in Table 1 (predictor variables) are for chi-square tests that were used to examine genotype differences for categorical variables. In Table 2 (cognitive tests), the p-values are for F tests that were used for the continuous cognitive variables. While there was a significant difference in the age distribution across *APOE *genotype categories, the difference was not significant when age was treated as a continuous variable (*F *= 2.65, *p *= 0.071). With the exception of RT, negative change scores indicate poorer performance at Wave 2. *E4 status did not increase the risk of head injury, problem drinking, stroke or current hypertension. There were no significant effects of *APOE *genotype on gender, education or premorbid intelligence.

**Table 1:** Descriptive statistics for predictor variables by *APOE *genotype

			*E4-/*E4-	*E4+/*E4-	*E4+/*E4+	
			(n = 1473)	(n = 510)	(n = 38)	
				
		*n*	*Freq (%)*	*Freq (%)*	*Freq (%)*	*p*
Age	60–62	1045	749 (71.7%)	282 (27.0%)	14 (1.3%)	**0.040**
	63–64	976	724 (74.2%)	228 (23.4%)	24 (2.5%)	
Gender	Male	1049	764 (72.8%)	267 (25.5%)	18 (1.7%)	0.837
	Female	972	709 (72.9%)	243 (25.0%)	20 (2.1%)	
Education	0–12 years	632	452 (71.5%)	167 (26.4%)	13 (2.1%)	0.907
	13 years	426	313 (73.5%)	107 (25.1%)	6 (1.4%)	
	14–15 years	207	156 (75.4%)	48 (23.2%)	3 (1.4%)	
	16+ years	756	552 (73.0%)	188 (24.9%)	16 (2.1%)	
Head injury	Yes	193	142 (73.6%)	47 (24.4%)	4 (2.1%)	0.941
	No	1828	1331 (72.8%)	463 (25.3%)	34 (1.9%)	
Problem drinking	Yes	468	349 (74.6%)	110 (23.5%)	9 (1.9%)	0.617
	No	1553	1124 (72.4%)	400 (25.8%)	29 (1.9%)	
Premorbid intelligence	Low	625	458 (73.3%)	159 (25.4%)	8 (1.3%)	0.599
(STW)	Medium	718	514 (71.6%)	190 (26.5%)	14 (1.9%)	
	High	654	481 (73.5%)	158 (24.2%)	15 (2.3%)	
Stroke or hypertension	Yes	1118	813 (72.7%)	285 (25.5%)	20 (1.8%)	0.911
	No	903	660 (73.1%)	225 (24.9%)	18 (2.0%)	
Stroke history	Yes	80	59 (73.8%)	19 (23.8%)	2 (2.5%)	0.882
	No	1941	1414 (72.8%)	491 (25.3%)	36 (1.9%)	
Current hypertension	Yes	1212	882 (72.7%)	309 (25.5%)	21 (1.7%)	0.804
	No	809	591 (73.1%)	201 (24.8%)	17 (2.1%)	

**Table 2:** Descriptive statistics for cognitive tests by *APOE *genotype

			*E4-/*E4-	*E4+/*E4-	*E4+/*E4+	
			(n = 1473)	(n = 510)	(n = 38)	
				
		*n*	*Mean (SD)*	*Mean (SD)*	*Mean (SD)*	*p*
Wave 2 SDMT		1984	49.55 (9.34)	49.71 (8.92)	48.00 (10.93)	0.547
Wave 2 immediate recall		1984	6.97 (2.22)	7.00 (2.09)	6.79 (2.74)	0.850
Wave 2 delayed recall		1984	6.18 (2.40)	6.12 (2.32)	5.89 (2.81)	0.704
Wave 2 digits backwards		1964	5.13 (2.21)	5.21 (2.22)	5.05 (1.80)	0.772
Wave 2 MMSE score		1974	29.24 (1.17)	29.25 (1.12)	28.71 (1.49)	**0.021**
Wave 2 simple RT (sec)		1925	0.28 (0.07)	0.28 (0.08)	0.26 (0.05)	0.659
Wave 2 choice RT (sec)		1919	0.33 (0.06)	0.33 (0.05)	0.32 (0.04)	0.902
						
Change in SDMT		1980	-0.99 (5.73)	-1.07 (5.60)	-1.63 (7.44)	0.781
Change in immediate recall		1984	-0.32 (2.07)	-0.29 (2.06)	-0.32 (2.49)	0.959
Change in delayed recall		1984	-0.17 (2.20)	-0.22 (2.25)	-0.21 (3.03)	0.907
Change in digits backwards		1962	0.09 (1.77)	0.30 (1.82)	0.27 (1.76)	0.075
Change in MMSE score		1971	-0.07 (1.16)	0.00 (1.17)	-0.27 (1.43)	0.274
Change in simple RT (sec)		1897	0.03 (0.06)	0.03 (0.07)	0.02 (0.05)	0.892
Change in choice RT (sec)		1880	0.01 (0.04)	0.01 (0.04)	0.01 (0.04)	0.838

As was expected, cognitive test scores declined significantly over the 4 year period: SDMT (Wave 1 mean 50.67; Wave 2 mean 49.62; *t*_1953 _= -8.14, *p *< .0001); Immediate Recall (Wave 1 mean 7.31; Wave 2 mean 6.98; *t*_1956 _= -7.00, *p *< .0001); Delayed Recall (Wave 1 mean 6.35; Wave 2 mean 6.16; *t*_1956 _= -3.83, *p *< .0001); Simple RT (Wave 1 mean 248 ms; Wave 2 mean 275 ms; *t*_1870 _= -19.37, *p *< .0001); Choice RT (Wave 1 mean 315 ms; Wave 2 mean 326 ms; *t*_1853 _= -11.99, *p *< .0001). MMSE deterioration was marginally significant (Wave 1 mean 29.29; Wave 2 mean 29.24; *t*_1946 _= -1.94, *p *= .052). Digits Backward performance improved (Wave 1 mean 5.00; Wave 2 mean 5.15; *t*_1956 _= -3.83, *p *< .0001). The observed effect sizes (Cohen's *d*) of the changes in the cognitive variables were .11 for SDMT, .14 for immediate recall, .07 for delayed recall, .07 for digits backwards, .05 for MMSE, .44 for simple RT and .23 for choice RT. There was one effect of *APOE *genotype on cognitive performance. At Wave 2, those homozygous for *E4 performed more poorly on the MMSE (*F *= 3.88, *df *= 2, *p *< .05). *APOE *genotype did not significantly predict change in performance on any of the cognitive tests.

Bivariate ANOVA models tested the interaction of *APOE *genotype and the other risk factors on cognitive change. Interaction effects of genotype on at least one cognitive test were found with education (delayed recall and digits backwards), head injury (SDMT) and premorbid intelligence (MMSE). In addition, the main effect of *APOE *genotype was significant for SDMT scores when adjusting for head injury and for digits backwards scores when adjusting for premorbid intelligence. No significant interaction effects of *APOE *with gender, current or past hazardous drinking, stroke or hypertension emerged. These five predictors were subsequently omitted from multivariate models.

### Multivariate models of the effects of genotype and risk factors on cognitive change scores

Table 3 shows the multivariate ANOVA models which examined the effect of the significant risk factors (education, head injury and intelligence) simultaneously. The findings were similar to the bivariate analyses. When accounting for the risk factors, *APOE *was found to be significantly associated with performance on SDMT and digits backwards. Interaction effects consistent with the bivariate analyses were also present for genotype and education (immediate and delayed recall), head injury (SDMT and MMSE) and premorbid intelligence (MMSE). All of the significant effect sizes were very small, with η^2 ^ranging from 0.003 to 0.009. R^2 ^for the models were also very small, ranging from 0.001 to 0.01.

**Table 3:** Multivariate models of change scores

	SDMT		Immediate recall		Delayed recall		Digits Backwards		MMSE		Simple RT		Choice RT	
	*F*	*p*	*F*	*p*	*F*	*p*	*F*	*p*	*F*	*p*	*F*	*p*	*F*	*p*
*APOE *Genotype	3.20	**0.041**	0.92	0.397	1.70	0.182	3.14	**0.044**	2.58	0.076	0.16	0.853	0.30	0.743
Education	2.09	0.099	0.86	0.461	0.32	0.812	1.61	0.185	2.49	0.059	0.08	0.971	0.51	0.674
Head injury	0.00	0.962	0.67	0.415	1.17	0.280	1.18	0.277	4.91	**0.027**	0.00	0.992	0.46	0.495
Premorbid intelligence	0.05	0.955	0.05	0.950	1.56	0.209	1.02	0.360	0.64	0.525	0.06	0.944	0.18	0.838
Genotype × Education	0.98	0.438	2.23	**0.038**	2.88	**0.008**	1.65	0.130	1.17	0.322	1.19	0.310	1.11	0.357
Genotype × Head injury	3.94	**0.020**	0.22	0.801	1.17	0.312	0.70	0.499	3.82	**0.022**	0.03	0.975	0.27	0.765
Genotype × Premorbid intelligence	0.47	0.757	1.04	0.388	2.32	0.054	1.07	0.370	2.71	**0.029**	2.04	0.087	0.35	0.842
Education × Head injury	1.72	0.160	1.04	0.375	0.12	0.951	1.26	0.285	2.68	**0.045**	0.55	0.650	0.78	0.504
Education × Premorbid intelligence	1.83	0.089	0.75	0.607	0.58	0.750	0.56	0.761	2.26	**0.035**	1.13	0.342	0.58	0.745
Head injury × Premorbid intelligence	0.25	0.778	1.18	0.307	0.06	0.941	0.97	0.379	0.13	0.879	0.83	0.435	1.38	0.253

**Figure 1:**
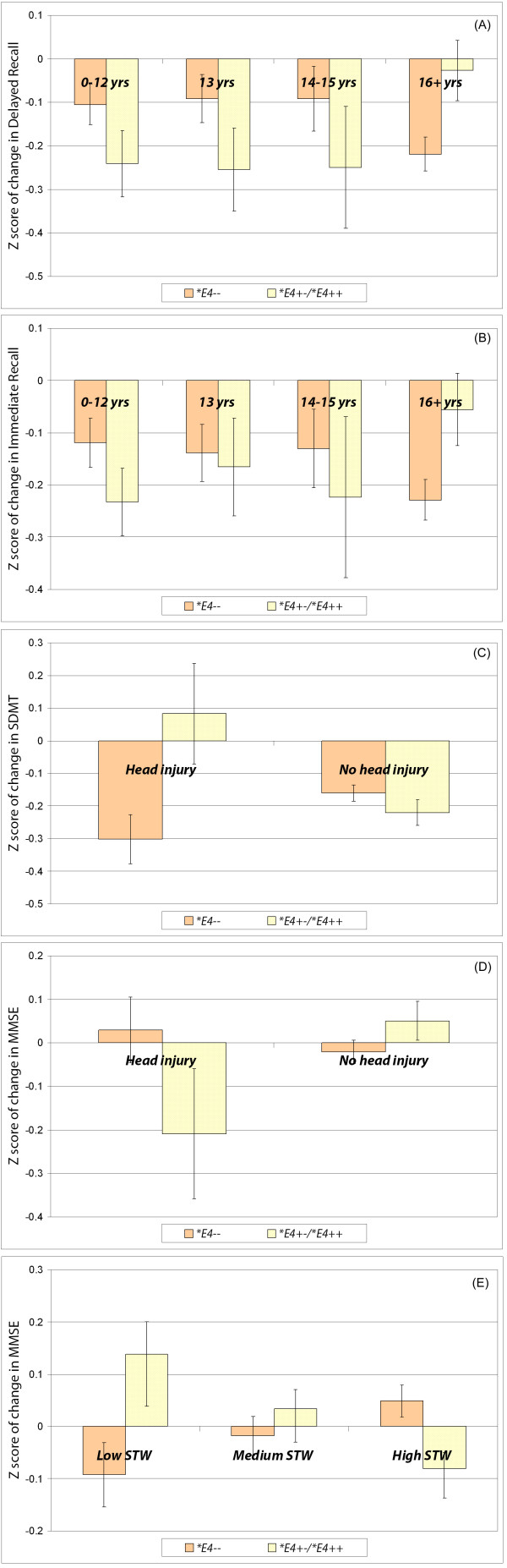
**Charts of significant interaction effects**. Panel A: Changes in Delayed Recall as a function of APOE genotype and education. Panel B: Changes in Immediate Recall as a function of APOE genotype and education. Panel C: Changes in SDMT as a function of APOE genotype and head injury. Panel D: Changes in MMSE as a function of APOE genotype and head injury. Panel E: Changes in MMSE as a function of APOE genotype and premorbid IQ. *Error bars represent standard error of the mean*.

**Bold **p-values represent p < .05;

Figure 1 illustrates these interaction effects, with *APOE *heterozygotes and homozygotes combined for simplicity. The interaction between *APOE *genotype and education was significant for both immediate and delayed recall change scores (Figure 1, Panels A and B). *E4 carriers with 16 or more years of education showed less memory decline than those with less education. However, among non-carriers, those who were more highly educated (16 years) tended to show more decline on the recall tasks than those with less education (0–15 years). There was a greater decrease in MMSE scores among *E4 non-carriers who experienced head injury (Figure 1, Panel D) than among *E4 carriers. Indeed, MMSE scores increased among carriers who reported head injury. SDMT scores, however, were greatly decreased among carriers who reported head injury, while SDMT scores remained virtually unchanged for carriers without head injury and non-carriers (Figure 1, Panel C). Premorbid intelligence had opposite effects for *E4 carriers and non-carriers on the change in their MMSE scores. Higher STW scores were associated with an MMSE decrease among carriers, while lower STW scores were associated with an MMSE decrease among non-carriers. This finding may be associated with ceiling effects on the MMSE (Figure 1, Panel E).

### Analysis excluding those clinically diagnosed with mild cognitive disorders or dementia

The analyses were rerun excluding the participants with a clinical diagnosis (n = 126). The proportion of participants with a diagnosis did not differ significantly across genotypes (χ^2 ^= 2.09, *df *= 2, *p *= .353). The main effect of *APOE *genotype on MMSE at Wave 2 remained significant. The multivariate model excluding participants with a clinical diagnosis is shown in Table 4. Most of the significant effects for the full sample were also found in this subanalysis.

**Table 4:** Multivariate models of change scores excluding mild cognitive disorders

	SDMT		Immediate recall		Delayed recall		Digits Backwards		MMSE		Simple RT		Choice RT	
	*F*	*p*	*F*	*p*	*F*	*p*	*F*	*p*	*F*	*p*	*F*	*p*	*F*	*p*
*APOE *Genotype	2.56	0.077	0.48	0.617	1.95	0.142	2.09	0.124	3.55	**0.029**	0.09	0.916	0.20	0.819
Education	2.83	**0.037**	0.94	0.422	1.13	0.337	1.53	0.206	3.20	**0.023**	0.25	0.863	0.61	0.605
Head injury	0.05	0.830	0.00	0.969	0.03	0.866	0.88	0.349	5.88	**0.015**	0.06	0.808	0.53	0.466
Premorbid intelligence	0.02	0.976	0.13	0.880	1.97	0.140	1.39	0.250	0.21	0.814	0.05	0.950	0.06	0.946
Genotype × Education	1.08	0.370	2.41	**0.025**	3.59	**0.002**	1.58	0.150	1.16	0.323	1.15	0.331	1.01	0.415
Genotype × Head injury	3.24	**0.039**	0.35	0.702	0.85	0.426	0.46	0.633	4.09	**0.017**	0.03	0.972	0.20	0.823
Genotype × Premorbid intelligence	0.59	0.667	0.90	0.462	1.84	0.119	1.09	0.360	1.26	0.284	2.95	**0.019**	0.40	0.806
Education × Head injury	2.24	0.081	0.80	0.495	0.16	0.922	1.38	0.246	3.22	**0.022**	0.37	0.774	0.26	0.852
Education × Premorbid intelligence	1.80	0.095	0.76	0.602	0.65	0.686	0.72	0.634	0.86	0.520	1.11	0.353	0.59	0.739
Head injury × Premorbid intelligence	0.44	0.647	0.45	0.639	0.35	0.708	0.92	0.398	0.02	0.982	0.58	0.557	0.95	0.387

**Bold **p-values represent p < .05; Underlined p-values indicate tests which change from significant to non-significant or *vice versa *when participants with mild cognitive disorders are excluded

## Discussion

The findings from the present analysis provide data on the association of APOE *E4 genotype and decline in cognitive performance over a four year period for individuals aged 65–69 years at follow-up. In comparison to Wave 1 results from this study where no effects were observed, we found that MMSE scores at Wave 2 were lower in *E4 homozygotes. Initial analyses showed no effect of genotype on cognitive change on any of the other cognitive tests. However, in further analyses, the main effect of APOE genotype was significant for SDMT when adjusting for head injury, and digits backward when adjusting for premorbid intelligence in further analyses. In addition, other significant interaction effects emerged in these multivariate models. Most of these interactions were in the predicted direction. Head injury was associated with greater decline in those with the APOE genotype when measured by the MMSE and fewer years of education were associated with greater cognitive decline for those homozygous or heterozygous for *E4 in association with the additional risk factor.

As expected, given the age of our sample, the findings of the present study are generally weaker than those from other population studies examining the effects of genotype. These studies usually report relatively robust associations between *E4 and cognitive change. For example, a study of 40 groups of USA-based Catholic clergy, aged 65 years or older (average age 76 years) reported that the *E4 allele was associated with faster decline on four cognitive domains over 6 years, with the effect greatest for memory [10]. Hofer et al. [8] reported faster rates of decline on tests of memory and speeded tests for those with an *E4 allele in an representative sample followed for 7 years, with the youngest aged 77 at follow-up. These findings remained when those with mild cognitive impairment were excluded. Similarly, Small, Basun and Backman [9] reported faster rates of decline on tests of recognition memory for those with *E4 compared to non-carriers within a sample of participants without dementia aged over 80 years. The weaker findings in our study are likely to due to the younger age of the participants who were mostly aged in their mid sixties.

The present study specifically sought to determine whether the young old age group might be at a stage when the effect of the APOE genotype 'kicked in'. Our findings are consistent with the effects of APOE emerging at this stage in those vulnerable as a result of the presence of an additional risk factor. We found interactions of genotype with education, intelligence and head injury, but not with high alcohol use, stroke or cardiovascular risk. Earlier longitudinal work reported the effects of genotype emerging in midlife in at risk individuals (those with a first degree relative with AD) [11,12] and under research conditions of high demand. However, given the few longitudinal studies of mid to early old population samples, and some inconsistent results from our own study, we believe that the hypothesis that the effects of the genotype emerge at this time requires further investigation.

Moreover, we consider that the positive findings we reported in the study need to be considered in the context of the number of non-significant findings on a range of tests, the limitations in power and the short follow-up period. As noted above, only one of seven cognitive tests showed an effect on performance at Wave 2. Moreover, the effects of the genotype on cognitive change were relatively weak, only emerging when models included potential risk factors. The interaction effects were not always consistent. Given these findings, it is important to consider whether the observed associations may have occurred by chance. Since a large number of potential interactions and effects were evaluated, the chance of obtaining a series of significant effects was high. However, measured against this consideration is the knowledge that, although the study is the largest of its type, the power of the study is relatively modest for detecting interaction effects between APOE *E4 and low prevalence risk factors such as head injury, stroke and hazardous alcohol use [1]. The tests used were standard neuropsychological tests which were not designed to measure performance under conditions of high cognitive demand. Moreover, the time period over which data are currently available is relatively short. With little cognitive change to work with, the study is (again) relatively underpowered. It is interesting to note that if the research had been guided by an exploratory rather than a hypothesis driven perspective, outcomes might arguably be adjusted for multiple testing, perhaps by imposing a p < 0.01 level of significance. If we had taken this approach, only a few findings would have emerged. However, those that meet this criterion are consistent with the evidence that episodic memory tasks are likely to be the ones most sensitive to *APOE *status (see for example Nilsson et al. [34]).

Whether these significant effects came about because of artifacts associated with psychometric properties of the tests used must be considered. The effects of *APOE *were present primarily for the MMSE. Reflecting its use as a screen for dementia, this test features a number of items that reflect orientation in time and location. It is assessing impairment at a relatively high threshold. However, a characteristic of the MMSE is its ceiling effect. This means that it is easier to register decline, but hard to show improvement. Models which recognize ceiling effects (effectively considering the scores to be censored above) involve very strong assumptions. We took the approach of including only decliners – those showing a decline on the MMSE – and repeating the analyses. The findings remained unchanged. We also examined the proportion of those decliners as a function of MMSE status, and found similar results. One clear implication is that these analyses warrant replication in four years when an additional wave of data will be available which tracks participants over a longer period into middle old age.

On balance, our interpretation of these findings is that effects of APOE *E4 in association with risk factors may begin to emerge after 60–65 years of age. The findings are consistent with biochemical research [13], and with previous studies indicating that the effects of *APOE *in the young old may only emerge in interaction with other risk factors and on tests sensitive to cognitive load [11,12]. We predict that these initial findings will be replicated more strongly at the next wave of the study.

## Conclusion

Our earlier study provided evidence that APOE *E4 status does not influence level of cognitive functioning for individuals aged 20–64 years. This suggested that cognitive aging processes are influenced by other unknown or uninvestigated factors at this age range or that the effect of *APOE *emerges at detectable magnitudes after 64 years of age. Four years later, follow-up data suggest that APOE *E4 is associated with poorer cognitive performance, as measured by the MMSE, and may influence the rate of cognitive decline in interaction with risk factors such as previous head injury or low education. It is possible that *E4 carriers appear to become vulnerable to greater cognitive decline in the presence of other risk factors at this age.

## Competing interests

The authors declare that they have no competing interests.

## Authors' contributions

HC drafted the manuscript and contributed to the design of the study; PJB drafted the manuscript and performed the analysis; AJM contributed to the design of the study, contributed to the analysis and revised the manuscript; AFJ revised the manuscript and contributed to the design of the study; HAM revised the manuscript and assisted with the APOE genotyping; KAM revised the manuscript and assisted with the APOE genotyping; KJA revised the manuscript and contributed to the design of the study; PSS revised the manuscript and contributed to the design of the study; SE assisted with the APOE genotyping; all authors read and approved the final manuscript.

## Pre-publication history

The pre-publication history for this paper can be accessed here:


